# Frequency of Screening and SBT Technique Trial - North American Weaning Collaboration (FAST-NAWC): a protocol for a multicenter, factorial randomized trial

**DOI:** 10.1186/s13063-019-3641-8

**Published:** 2019-10-11

**Authors:** K. E. A. Burns, Leena Rizvi, Deborah J. Cook, Andrew J. E. Seely, Bram Rochwerg, Francois Lamontagne, John W. Devlin, Peter Dodek, Michael Mayette, Maged Tanios, Audrey Gouskos, Phyllis Kay, Susan Mitchell, Kenneth C. Kiedrowski, Nicholas S. Hill, For the Canadian Critical Care Trials Group

**Affiliations:** 1 Interdepartmental Division of Critical Care, University of Toronto, Toronto, ON Canada; 2 Department of Medicine, Division of Critical Care Medicine, St Michael’s Hospital, 30 Bond Street, Office 4-045 Donnelly Wing, Toronto, ON M5B 1W8 Canada; 3 Li Ka Shing Knowledge Institute, St. Michael’s Hospital, 30 Bond Street, Office 4-045 Donnelly Wing, Toronto, ON M5B 1W8 Canada; 4 Department of Health Research Methods, Evidence and Impact, McMaster University, Hamilton, ON Canada; 5 Ottawa Hospital Research Institute, University of Ottawa, Ottawa, ON Canada; 6 Department of Medicine, McMaster University, Hamilton, ON Canada; 7 Centre de Recherche du Centre Hospitalier Universitaire de Sherbrooke, Sherbrooke, QC Canada; 8 School of Pharmacy, Northeastern University, Boston, MA USA; 9 Division of Pulmonary, Critical Care and Sleep Medicine, Tufts Medical Center, Boston, MA USA; 10 Centre for Health Evaluation and Outcome Sciences, Vancouver, BC Canada; 11 University of British Columbia, Vancouver, BC Canada; 12 Critical Care Medicine, Longbeach Memorial, Longbeach, CA USA; 13 Patient and Family Advisory Committee Member, FAST – NAWC Trial, Toronto, Canada

**Keywords:** Weaning, Spontaneous breathing trial, Screening, Randomized controlled trial, Successful extubation

## Abstract

**Rationale:**

In critically ill patients receiving invasive mechanical ventilation (MV), research supports the use of daily screening to identify patients who are ready to undergo a spontaneous breathing trial (SBT) followed by conduct of an SBT. However, once daily (OD) screening is poorly aligned with the continuous care provided in most intensive care units (ICUs) and the best SBT technique for clinicians to use remains controversial.

**Objectives:**

To identify the optimal screening frequency and SBT technique to wean critically ill adults in the ICU.

**Methods:**

We aim to conduct a multicenter, factorial design randomized controlled trial with concealed allocation, comparing the effect of both screening frequency (once versus at least twice daily [ALTD]) and SBT technique (Pressure Support [PS] + Positive End-Expiratory Pressure [PEEP] vs T-piece) on the time to successful extubation (primary outcome) in 760 critically ill adults who are invasively ventilated for at least 24 h in 20 North American ICUs. In the OD arm, respiratory therapists (RTs) will screen study patients between 06:00 and 08:00 h. In the ALTD arm, patients will be screened at least twice daily between 06:00 and 08:00 h and between 13:00 and 15:00 h with additional screens permitted at the clinician’s discretion. When the SBT screen is passed, an SBT will be conducted using the assigned technique (PS + PEEP or T-piece). We will follow patients until successful extubation, death, ICU discharge, or until day 60 after randomization. We will contact patients or their surrogates six months after randomization to assess health-related quality of life and functional status.

**Relevance:**

The around-the-clock availability of RTs in North American ICUs presents an important opportunity to identify the optimal SBT screening frequency and SBT technique to minimize patients’ exposure to invasive ventilation and ventilator-related complications.

**Trial registration:**

Clinical Trials.gov, NCT02399267. Registered on Nov 21, 2016 first registered.

**Electronic supplementary material:**

The online version of this article (10.1186/s13063-019-3641-8) contains supplementary material, which is available to authorized users.

## Introduction

Weaning from invasive mechanical ventilation (MV) is the process during which the work of breathing is transferred from the ventilator back to the patient. Nearly 40% of the time spent on invasive MV is dedicated to weaning [1, 2]. Although invasive MV is effective in managing respiratory failure, its use is associated with the development of numerous complications including ventilator-associated pneumonia (VAP) and respiratory muscle weakness [3]. The risk for VAP increases after the fifth day of invasive MV, is associated with substantial morbidity, and may increase mortality [4]. Conversely, premature or failed attempts at extubation necessitating reintubation are also associated with greater risk of VAP [5], prolonged intensive care unit (ICU) stay, and increased mortality [6, 7]. Consequently, in their efforts to minimize patient’s exposure to invasive MV, clinicians are challenged by a “trade-off” between the complications associated with protracted ventilation and the risks associated with failed attempts at extubation [8].

More than two decades of research support the use of specific strategies to limit invasive MV including: (1) the use of multidisciplinary screening protocols to identify appropriate candidates for a spontaneous breathing trial (SBT) [9, 10]; (2) the conduct of SBTs [6, 7, 11, 12] in patients who pass screening criteria; and (3) the use of specific modes and techniques (reductions in Pressure Support [PS]) and once daily (OD) SBTs (PS ± positive end expiratory pressure [PEEP] or T-piece) [12–14] to discontinue ventilator support in patients who fail an initial SBT. In a 2014 Cochrane review of 17 RCTs (*n* = 2434), use of an SBT screening protocol compared to usual care was associated with significant reductions in weaning time, duration of MV, and ICU stay [9]. However, the strength of the conclusions that could be made from this meta-analysis were limited by heterogeneous populations, individual study risk of bias, and comparison of OD screening (intervention arm) to usual care (control arm) in most included trials. Importantly, no trial in this review compared more frequent screening to daily screening. Only one trial (*n* = 385) compared twice daily screening to usual care and noted a significantly shorter duration of MV and a trend toward a lower VAP with twice daily screening [15]. In national and international weaning surveys, daily SBT screening is the current standard of care [16, 17]. Notwithstanding, daily screening may be poorly aligned with the continuous care provided in most ICUs because it is not patient-centered and disregards the impact that treatment interventions, initiated after morning patient care rounds (e.g. reducing sedation), may have on SBT screening efforts later in the day.

The preferred technique to conduct an SBT remains unclear. Although PS SBTs are more commonly used in North America, significant inter-institutional variability exists in how SBTs are conducted [18]. Two meta-analyses of randomized controlled trials (RCTs) compared PS and T-piece weaning, including, but not limited to, the conduct of SBTs, found beneficial effects of PS weaning [19, 20]. Similarly, a meta-analysis that directly compared alternative SBT techniques [11] and two guidance documents [12, 21] support use of PS SBTs. Conversely, a physiologic meta-analysis favored use of T-piece SBTs [22].

Although a large body of evidence regarding weaning and SBT conduct exists, it remains insufficient to guide care regarding how frequent SBT screening should occur and the SBT technique that should be used. The around-the-clock availability of respiratory therapists (RTs) in most North American ICUs presents a unique opportunity to identify the optimal SBT screening frequency and SBT technique. In the Frequency of Screening and SBT Technique – North American Weaning Collaborative (FAST-NAWC) Trial, we will compare the effect of different screening frequencies (OD vs at least twice daily [ALTD]) and SBT techniques (PS + PEEP vs T-piece) in critically ill adults on time to successful extubation [23].

## Objectives

### Primary objectives

The primary objectives of the FAST-NAWC trial are to demonstrate the effect of the alternative:

1. different screening frequencies (OD vs ALTD) on time to successful extubation;
2. different SBT techniques (PS + PEEP vs T-piece) on time to successful extubation.

### Secondary objectives

We will obtain estimates of the impact of the alternative screening and SBT techniques on other clinically important outcomes (see “Secondary outcomes” below).

## Methods

### Study population

We will include 760 critically ill adults aged ≥ 18 years (USA) or ≥ 16 years (Canada) or admitted to an adult ICU in approximately 20 ICUs in North America.

### Eligibility

We will include critically ill adults who: (1) have received invasive mechanical ventilation for ≥ 24 h; (2) are capable of initiating *spontaneous* breaths or *triggering* the ventilator to give a breath on ventilator modes commonly used in the ICU; (3) require a fractional concentration of inspired oxygen (FiO_2_) ≤ 70%; and (4) PEEP ≤ 12 cm H_2_O. We will exclude patients who meet one or more of the exclusion criteria listed in Table 1.

**Table 1:** Exclusion criteria

1. Brain death or expected brain death	
2. Patients who have evidence of myocardial ischemia in the 24-h period before enrollment, except if current trend in troponin is downward AND it has been ≥ 24 h since last troponin peak or the patient has undergone a revascularization procedure and attending physician has no concerns regarding ongoing ischemia	
3. Patients who have received continuous invasive mechanical ventilation for ≥ 2 weeks	
4. Patients who have a tracheostomy in situ at the time of screening	
5. Patients who are receiving sedative infusions for seizures or alcohol withdrawal	
6. Patients who require escalating doses of sedative agents	
7. Patients who are receiving neuromuscular blockers or who have known quadriplegia, paraplegia, or four-limb weakness or paralysis preventing active mobilization (e.g. active range of motion, exercises in bed, sitting at edge of bed, transferring from bed to chair, standing, marching in place, ambulating)	
8. Patients who are moribund (e.g. at imminent risk for death) or who have limitations of treatment (e.g. withdrawal of support, do not reintubate order, however, do not resuscitate orders will be permitted)	
9. Patients who have profound neurologic deficits (e.g. after cardiac or respiratory arrest, large intracranial stroke or bleed) or GCS ≤ 6	
10. Patients who are using modes that automate SBT conduct	
11. Patients who are current enrolled in a confounding study that includes a weaning protocol, or	
12. Patients who were previously enrolled in this trial	
13. Patients who have already undergone an SBT or are on T-piece, or CPAP alone (without PS), or PS ≤ 8 cm H_2_O regardless of PEEP, or other “SBT equivalent” settings immediately before randomization	
14. Patients who have already undergone extubation (planned, unplanned [e.g. self, accidental]) during the same ICU admission	

*PS* Pressure Support, *PAV* Proportional Assist Ventilation, *AC* assist control, *SIMV* synchronized intermittent mandatory ventilation, *PRVC* pressure regulated volume control, *VS* volume support, *APRV* airway pressure release ventilation, *FiO*_*2*_ inspired fractional concentration of oxygen, *PEEP* positive end-expiratory pressure, *GCS* Glasgow Coma Scale, *SBT* spontaneous breathing trial, *CPAP* continuous positive airway pressure

### Enrollment

**Fig. 1:**
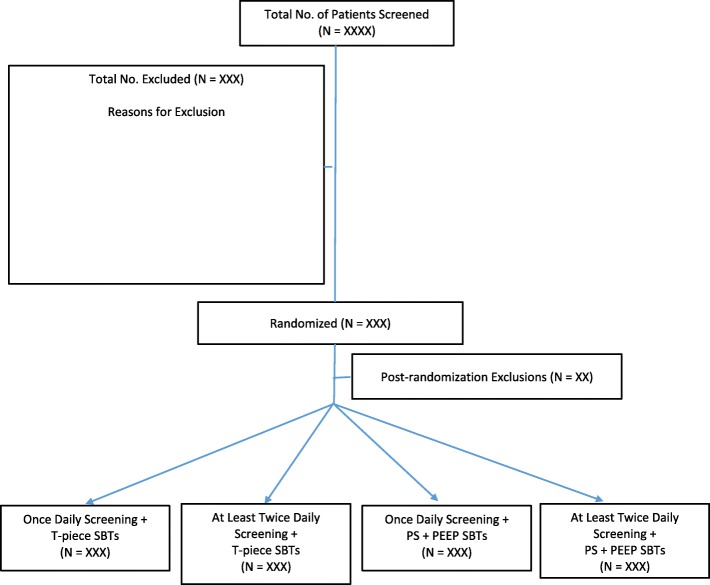
Cumulative hazard and survival functions of patients infected by K. pneumoniae fitted to Lognormal distribution

Research personnel (research coordinators and/or RTs) will identify, consent, and enroll eligible patients from Monday to Friday during regular hours using a central randomization system, stratified by ICU with variable undisclosed block sizes. With the factorial design, patients will be randomized to both a screening frequency (OD vs ALTD) and an SBT technique (PS + PEEP vs T-piece) (Fig. 1).

### Informed consent

This protocol was approved by the research ethics board of St. Michael’s Hospital (Toronto) and of participating ICUs. Given the minimal risk associated with the interventions being evaluated and the need to enroll patients as soon as possible after they can either initiate spontaneous breaths or trigger breaths, we will request ethics approval to use a hybrid consent model that prioritizes obtaining consent from patients (with decision-making capacity) or SDMs (when available) and permits deferred consent in their absence. For patients who are enrolled under deferred consent [24], research personnel will obtain consent as soon as possible after randomization. We have used this hybrid consent model in two multicenter, pilot, screening frequency trials comparing OD and ALTD screening [25].

### Study interventions

#### Screening for readiness to undergo a spontaneous breathing trial

In the OD arm, RTs will screen study patients daily between 06:00 and 08:00 h. In the ALTD arm, patients will be screened at least twice daily between 06:00 and 08:00 h and between 13:00 and 15:00 h; additional screening will be permitted at the discretion of the ICU team. If a screening period is missed (inadvertently or due to an operation/procedure necessitating absence from the ICU), it may be conducted later on the same day and ideally within 6 h of the scheduled screening period. For patients randomized *after* 10:00 h, only one screening assessment will be required on the first study day regardless of study arm.

To pass a screening assessment and undergo an SBT, all of the following criteria must be met:

1. The patient must be capable of initiating spontaneous breaths on PS or Proportional Assist Ventilation (PAV) or triggering breaths on volume or pressure Assist Control (AC), volume or pressure synchronized intermittent mandatory ventilation (SIMV) ± PS, pressure regulated volume control (PRVC), volume support (VS), or Airway pressure regulated volume (APRV);
2. The ratio of partial pressure of oxygen to FiO_2_ (PaO_2_/FiO_2_) ≥ 200 mmHg;
3. Respiratory rate ≤ 35 breaths/min;
4. PEEP ≤ 10 cm H_2_O;
5. Heart rate ≤ 140 beats/min;
6. The ratio of respiratory frequency to tidal volume (f/VT) < 105 breaths/min/L [26] during a 2 min assessment on Continuous positive airway pressure (CPAP) of 0 cm H_2_O (alternatively PS = 0 cm H_2_O /PEEP = 0 cm H_2_O).

#### Conduct of spontaneous breathing trials

After passing a screening assessment, patients will undergo an initial SBT according to treatment assignment (PS + PEEP vs T-piece). All SBTs will be 30–120 min in duration with the actual duration selected by clinicians [7, 21]. SBTs will be conducted on T-piece (off ventilator with no CPAP/PEEP) or with PS > 0 and ≤ 8 cm H_2_O with PEEP > 0 and ≤ 5 cm H_2_O [21]. Between SBT trials, patients will be returned to the mode of ventilation used before the SBT, unless criteria are met to remain on/return to a mode of support that assumes no spontaneous or triggered breaths (Additional file 1). We will use standardized criteria to determine SBT failure in both arms [27] (Table 2). After an unsuccessful SBT, patients will be returned to the ventilator settings used before the SBT and ventilator settings will be adjusted to restore respiratory comfort.

**Table 2:** Criteria for spontaneous breathing trial failure

A failed SBT will be defined by the presence of any ONE of:	
(1) A respiratory rate > 35 breaths/min with signs of respiratory distress or an increase in respiratory rate ≥ 20% from baseline with signs of respiratory distress	
(2) Oxygen saturation of arterial blood (SaO_2_) or pulse oximetry < 90%	
(3) Heart rate > 140 beats/min with signs of respiratory distress or an increase in HR ≥ 20% from baseline with signs of respiratory distress	
(4) Systolic blood pressure ≥ 180 or ≤ 90 mmHg	
(5) The presence of somnolence, agitation, diaphoresis, or anxiety	
(6) Requirement for the addition of or an increase in vasopressor or inotropic agent support	
(7) Chest pain or other limiting pain precluding further continuation	

#### Criteria to suspend the protocol and return to a controlled/supported mode of ventilation

Patients should remain on a mode that permits spontaneous or triggered breaths between SBTs and at night. In all groups, patients will be permitted to return to/remain on a supported mode of ventilation without spontaneous or triggered breaths when one or more criteria are met (Table 3). Patients who meet any criteria will be reassessed daily to identify the earliest time when they meet initial inclusion criteria and the screening and SBT protocols can be resumed.

**Table 3:** Criteria to suspend the protocol and return to a controlled/supported mode of ventilation

(1) Surgery or invasive procedures requiring sedation	
(2) Respiratory distress as defined by:	
a) sustained hypoxemia (pulse oximetry oxygen saturation [SpO_2_] < 90%) with an FiO_2_ > 60% and PEEP > 10 cm H_2_O or hypercapnia with pH < 7.30 OR clinical respiratory distress	
b) repeated episodes (≥ 3 episodes within 1 h) wherein an inspiratory pressure (drive pressure + PEEP on pressure modes or plateau pressure on volume modes) of 35 cm H_2_O or more is attained (despite suctioning, bronchodilation, etc.)	
(3) Hemodynamic instability despite fluid boluses and requirement for high dose vasopressors: norepinephrine > 15 μg/min (0.2 μg/kg/min) or equivalent	
(4) Suspected myocardial ischemia based on EKG and/or elevated Troponin I	
(5) Neurologic deterioration with need to control PaCO_2_ (e.g. raised intracranial pressure) or central hypoventilation	
(6) Respiratory rate < 10 breaths/min related to need for increased sedation	
(7) PEEP ≥ 13 cm H_2_O	
(8) FiO_2_ ≥ 71%	

*FiO*_*2*_ inspired fractional concentration of oxygen, *PEEP* positive end-expiratory pressure, *EKG* electrocardiogram

### Extubation

Patients who pass an SBT will be assessed for extubation. Extubation should be performed as soon as possible after passing an SBT. To be extubated patients should meet all criteria depicted in Table 4 [27].

**Table 4:** Extubation criteria

(1) SpO_2_ ≥ 90% or at baseline level in chronically hypoxemic patients on an FiO_2_ ≤ 40% and PEEP ≤ 5 cm H_2_O	
(2) A cough of sufficient strength to clear secretions and must not require suctioning more than every 2 h	
(3) Patients should be hemodynamically stable (off vasopressors or on minimal levophed, i.e. ≤ 7 μg/min [0.1 μg/kg/min or equivalent])	
(4) A level of consciousness sufficient to ensure airway protection and	
(5) A cuff leak is present	
All of the above criteria (except nos. 4 and 5) will also apply to patients who undergo trach mask trials and are disconnected.	

*SpO*_*2*_ pulse oximetry saturation, *FiO*_*2*_ fraction of inspired oxygen concentration, *PEEP* positive end-expiratory pressure

As this was not a trial focused on extubation, we did not protocolize extubation. Conversely, we will record the time that patients met criteria for extubation and the time patients were actually extubated.

### Other important considerations

We standardized approaches to ventilator titration, use of NIV after extubation, reintubation, and tracheostomy [28, 29] (Additional file 1).

### Follow-up

Successful extubation is defined as the time when unsupported, spontaneous breathing began and was sustained for ≥ 48 h after extubation/disconnection in patients with tracheostomy [27]. We will collect daily data up to successful extubation, ICU death, ICU discharge, or until day 60 after randomization (deemed ventilator-dependent), whichever comes first. All patients will be followed to hospital discharge.

### Study outcomes

#### Primary outcome

The primary outcome will be the time to successful extubation.

#### Secondary outcomes

Secondary outcomes will include: (1) ICU mortality; (2) hospital and 90-day mortality [30]; (3) time to first passing an SBT; (4) total duration of mechanical ventilation (invasive and non-invasive); (5) ICU length of stay; (6) hospital length of stay; (7) use of NIV after extubation [15, 31]; (8) VAP; (9) adverse events (AEs), self-extubation, tracheostomy [28, 29], reintubation, prolonged ventilation (patients who remain intubated at day 14 and/or day 21), ICU readmission [32, 33]; (10) proportion of patients who receive sedation, analgesia, antipsychotics at key time points; (11) proportion who screen positive for delirium at key time points [34–37], (12) HRQoL (EuroQuol EQ-5D) six months after randomization [38, 39]; and (13) functional status six months after randomization using the Functional Independence Measure (FIM) [40, 41].

### Analytic plan

We will summarize baseline data using descriptive statistics.[42]. All analyses will be performed adhering to the intention-to-treat principle.

### Primary analysis

Time-to event outcomes present special challenges because death is a competing risk and survivor-only analyses are improper sub-groups. We will construct cumulative incidence curves to provide outcome estimates accounting for death for screening frequency and SBT technique. Cause-specific treatment effects will be depicted with hazard ratios (HR) with 95% confidence intervals (CI) from Cox models.

### Secondary analysis

We will report treatment effects in time to event analyses using HR and odds ratio (OR) with 95% CIs for binary outcomes and mean difference with 95% CI for continuous outcomes [42].

### Exploratory and adjusted analyses

To assess the effect of age (continuous variable) by treatment interaction on the HR of time to successful extubation, we will construct a Cox regression model using a restricted cubic spline for age. Instead of arbitrarily assigning different levels for each age group, period, and cohort, we will create a smoothing function or spline (collections of cubic polynomials joined smoothly at a predefined number of points [knots]). The number of knots is expected to be between three and five but will be selected based on the sample size assuming that the relationship with age will change gradually and smoothly. We will evaluate fit using bootstrap techniques. This technique allows for non-linearities and interactions between variables that are more flexible than the linear contrasts traditionally used in regression models and is easier to depict and interpret [43]. In exploratory and adjusted analyses, we will assess for an interaction between screening frequency and SBT technique and variables (e.g. COPD, frailty etc.) of potential prognostic importance.

### Interim analyses

Interim analyses for safety (AEs) and efficacy (primary outcome) will be performed at 25%, 50%, and 75% of accrual and reviewed by the Data Safety and Monitoring Committee (DSMB). Given the potential risk of stopping early for benefit, statistical significance will be declared using small *p* values according to the O’Brien-Fleming boundaries for the primary outcome and reintubation rate [44].

### Sample size

To compute sample size and take into consideration deaths that occur before successful extubation, we used cumulative incidence curves generated from our pilot trials and computed three mortality HRs per patient day (HR 2.9 [Release Trial] and HR 3.3 [SENIOR Trial] and HR 3.2 [combined]) [25]. We will require 760 patients to demonstrate a reduction in time to successful extubation from a median of 5.0 days to 4.0 days (HR 1.25) [27, 45] with 80% power and α = 0.05 and allowing for three interim analyses. A priori, we do not expect an interaction since mechanistically and sequentially an interaction is unlikely. Since the groups are orthogonal, the main effects (in the absence of interactions) will have the same power to detect the same size differences.

### Trial organization

The Applied Health Research Centre (AHRC; www.ahrconline.com) of St. Michaels Hospital will serve as the data management and coordinating center. The AHRC is a not-for profit academic research organization affiliated with the University of Toronto. The Methods Centre will be responsible for data management and analysis as well as providing progress and data reports to the Steering Committee and DSMB.

The FAST-NAWC is being implemented with the input of ICU survivors and family members of former ICU patients from Canada and the USA. Our Patient and Family Advisory Committee (PFAC) members have direct experience with mechanical ventilation. The PFAC will serve in an advisory capacity to the Steering Committee. During protocol development, our PFAC members aided in identifying our primary outcome and advocated to include a six-month follow-up study. All PFAC members provided letters of support for grant submissions and one PFAC member reviewed a portion of the grant [46]. During trial implementation, PFAC members will: (1) be represented on the trial Steering Committee and DSMB; (2) directly advised the Steering Committee on study design and implementation issues; (3) assist with preparation of study materials; (4) aid in selecting a metric to assess functional status at six-month follow-up assessments; (5) participate in bimonthly teleconferences; and (6) contribute to developing a moderated on-line space that will serve as a repository for patient and family narratives [46].

### Trial status

The FAST PILOT trial was launched on 15 June 2016 in 11 ICUs and was completed on 8 December 2018 [47]. After received full funding to conduct the FAST NAWC (protocol version 5; 4 May 2018), we re-launched the factorial design RCT on 18 January 2018. We anticipate recruitment will be completed by March 2021.

## Discussion

The FAST-NAWC Trial is novel in several ways. First, the FAST-NAWC Trial will be the largest weaning trial conducted in the North America where weaning involves close collaboration between RTs and physicians. Second, the FAST-NAWC seeks to identify the optimal SBT screening frequency and SBT technique to minimize patients’ exposure to invasive ventilation and ventilator-related complications. Third, this trial is being implemented with novel collaborations between Canadian and American research networks and respiratory care and critical care societies. Finally, the FAST-NAWC Trial is being conducted with the input of ICU survivors and family members. They will ensure that the trial is implemented in a manner that is sensitive to patient’s and families’ needs.

To address the concerns that elderly, critically ill patients may experience more AEs with more frequent SBT screening and be less likely to be enrolled in this trial due to concomitant treatment limitations and co-morbidities, we conducted two parallel, multicenter, pilot screening frequency trials [25]. Both trials compared OD with ALTD screening in elderly patients aged ≥ 65 years (SENIOR Trial; ClinicalTrials.gov NCT02243449; 11 ICUs) and non-elderly patients aged < 65 years (RELEASE Trial; NCT02001220; 10 ICUs) [25]. Recognizing the need to change screening culture, we prioritized the evaluation of SBT screening frequency and permitted centers to use their preferred SBT technique in both pilot trials. We demonstrated similar recruitment and consent rates, few AEs, and comparable outcomes in younger and older patients [25]. Conduct of the pilot trials enabled us to refine several exclusion criteria and enhance the generalizability of our findings. Subsequently, we conducted a factorial design, FAST pilot trial (*n* = 110) comparing both screening frequency and SBT technique [48]. With this trial, we refined recruitment estimates, identified barriers to recruitment, and assessed potential co-interventions. Patients enrolled in the FAST pilot trial will be included in the FAST-NAWC trial.

Careful consideration has been given to important aspects of the FAST-NAWC study design to limit selection, identification, treatment, and performance bias in this necessarily unblinded weaning trial. To limit selection bias, we will use a central randomization process with full allocation concealment. To limit identification bias, RTs will conduct SBT screening assessments and SBTs. To limit delays in identifying SBT candidates, we avoided use of subjective assessments in selecting our SBT screening criteria (e.g. level of consciousness, no sedation, no vasopressors, hemoglobin > 100 g/L). Conversely, we included an objective test, the rapid shallow breathing index (f/VT) [26], measured on standardized settings, in all SBT screening assessments. We will record practices before SBT screening that have the potential to influence SBT performance including pain, sedation, and delirium management and whether patients are being mobilized. To enhance the generalizability of our findings, we will permit SBTs to be 30–120 min in duration at clinician’s discretion. Finally, to limit treatment bias, we provided guidance on: (1) titration of ventilator settings, PEEP, and FiO_2_; (2) use of NIV after extubation; (3) reintubation; and (4) tracheostomy.

Establishing the optimal screening frequency and SBT technique is appealing to ICU clinicians because these interventions are sensible, low-risk, and represent an efficient use of current resources. Findings from this trial have the potential to change clinical practice, enhance the care delivered to critically ill adults, and improve outcomes.

## Additional file

Additional file 1: Additional Protocol Information. (DOCX 25 kb)

## Data Availability

There are no data related to the protocol manuscript.
