# Supplementary material for: Frequency of Screening and SBT Technique Trial - North American Weaning Collaboration (FAST-NAWC): a protocol for a multicenter, factorial randomized trial
Source: Trials. 2019 Oct 11;20:587. doi: 10.1186/s13063-019-3641-8 (PMC6787986; doi:10.1186/s13063-019-3641-8)
Supplement: Supplementary file 1 — Additional Protocol Information. (DOCX 25 kb) [file 13063_2019_3641_MOESM1_ESM.docx]

**FAST NAWC**

**(i) Mechanical Ventilation Titration (Following Randomization):**

*Ventilator Titration:* Patients will preferentially remain on a mode that permits spontaneous or triggered breaths between SBTs and at night. While on study protocol, we suggest that RTs approach the ventilator at least every 4 to 6 hours to assess and titrate ventilator settings in PS mode (preferred), FiO_2,_ and PEEP levels.

We suggest:

- The level of ventilator support is reevaluated **at least every 4 - 6 hrs** and titrated to avoid respiratory distress (use of accessory muscles or RR > 30 breaths/min (alternatively, > 34 breaths/min in patients with COPD or a central neurologic disease) or overassistance.
- PEEP and FiO_2_ should be reevaluated **at least every 4-6 hrs** and will be titrated to maintain PaO_2_ 60- 80 mm Hg (or at baseline levels in hypercarbic patients) or SpO_2_ 90 – 95%. **SpO_2_ will be assessed at least every 4 - 6 hours** and arterial blood gases will be performed at the discretion of the RT/physician.

**(ii) Guidance on Noninvasive Positive Pressure Ventilation Initiation**

*Use of NIV after extubation:* We developed initiation criteria to guide the use of NIV after extubation. For NIV to be used after extubation, patients must have successfully completed an SBT and been extubated. In the WE Succeed Trial, patients cannot be extubated to NIV to facilitate weaning without previously passing an SBT as this technique obviates the need for an SBT. While a meta-analysis by our group supports extubating selected patients (especially those with COPD) directly to NIV, our survey supports that few clinicians extubate patients to NIV in practice [19,43].

To Initiate NIV: We suggest that the presence of any two of the following criteria be used to initiate NIV

(i) clinical signs of respiratory distress/muscle fatigue and RR > 30 breaths/min or a 50% increase in RR from baseline,

(ii) respiratory acidosis (pH < 7.35 with PaCO_2_ > 45 mmHg),

(iii) hypoxemia (SpO_2_ < 90% or PaO_2_ < 60mmHg with FiO_2_ > 50%)

Initiation Technique: NIV is delivered with the patient in a seated position and the upper body elevated at a minimum of 30°. The patient-ventilator interface is fitted to the patient with oronasal masks preferred over nasal masks. Patients are encouraged to hold the mask up to their face before securing it with head straps. Excessive tension on the straps is avoided. A nasal hydrocolloid protection dressing is used to minimize discomfort related to mask application. Initial breaths are made with inspiratory pressure of 8 cm H_2_O and no expiratory pressure. In the first 2 - 5 minutes, progressive increases in inspiratory and expiratory pressure are made in increments of 2 cm H_2_O. PEEP (or expiratory positive airway pressure) can be increased to a maximum of 3 cm H_2_O in COPD patients and 10 cm H_2_O in hypoxemic patients and for treatment of atelectasis. Heated humidification is preferentially used in patients, especially those with persistent hypercapnia. A variety of interfaces (different shapes/sizes) are available to minimize leaks and ensure patient comfort. Leaks and asynchronies are minimized.

Regardless of treatment assignment, the total duration of mechanical ventilation will include the time spent on NIV after extubation prior to achieving successful extubation.

**(iii) Guidance for Reintubation and Reinitiation of Invasive Weaning**

*Reintubation:* Patients requiring reintubation after successful extubation will be ventilated according to usual practice and at the discretion of the clinical team. If patients are reintubated within 48 hours of extubation, they will be reassessed daily to identify the earliest time when they can initiate spontaneous breaths (PS or PAV) or trigger breaths (volume or pressure AC, or volume or pressure SIMV ± PS, or PRVC or APRV) and whether they meet study initial inclusion criteria so that the assigned screening frequency and SBT technique can resume.

We suggest that the following criteria be used to guide intubation. Intubation should be considered if at least one major criterion or two minor criteria are met:

**Reintubation Major criteria:** (i) cardiac arrest, (ii) respiratory pauses with loss of consciousness or gasping for air, (iii) psychomotor agitation interfering with nursing care, (iv) heart rate < 50 bpm with loss of alterness or (v) hemodynamic instability with systolic pressure < 80 mmHg for > 30 minutes despite adequate volume challenge, use of vasopressors or both.

For patients on NIV: (vi) a change in mental status (decrease in LOC, severe agitation) rendering the patient unable to tolerate NIV, (vii) persistent or worsening signs of respiratory distress/muscle fatigue despite NIV, (viii) abundant secretions that cannot be effectively cleared or are associated with acidosis, hypoxemia or a change in mental status with NIV, (ix) failure to improve pH or PaCO_2_ with NIV.

**Minor criteria:** (i) RR > 35 breaths/min and exceeding RR after extubation, (ii) pH < 7.30 and less than the value after extubation, (iii) sustained decrease in SpO_2_ < 85% despite high FiO_2_ (≥0.80) or (iv) decreasing level of consciousness (deterioration from baseline).

**(iv) Guidance for Tracheostomy and Conduct of Trach Mask Trials**

*Tracheostomy:* Patients who do not have a tracheostomy at study inclusion may receive a tracheostomy during their care. We suggest that investigators, when possible, wait until at least day 10 before considering an elective tracheostomy [44,45]. Physicians may offer tracheostomy earlier if it is not an elective procedure (e.g., fixed airway obstruction).

**Conduct of Tracheostomy Mask Trials**

- Following a tracheostomy, patients will undergo Trach Mask Trials of increasing duration and frequency in both treatment arms titrated to avoid respiratory distress (use of accessory muscles or RR > 35 breaths/min
- Patients will be reevaluated at least every 4 - 6 hrs while awake to undergo Trach Mask Trials.
- Disconnection from tracheostomy will follow after patients meet criteria for disconnection
